# Diversity of Vascular Niches in Bones and Joints During Homeostasis, Ageing, and Diseases

**DOI:** 10.3389/fimmu.2021.798211

**Published:** 2021-12-17

**Authors:** Naveen Kumar, Pepijn Saraber, Zhangfan Ding, Anjali P. Kusumbe

**Affiliations:** Nuffield Department of Orthopaedics, Rheumatology and Musculoskeletal Sciences (NDORMS), Tissue and Tumor Microenvironments Group, University of Oxford, Oxford, United Kingdom

**Keywords:** bone, joint, ageing, endothelial cell, vascular niche

## Abstract

The bones and joints in the skeletal system are composed of diverse cell types, including vascular niches, bone cells, connective tissue cells and mineral deposits and regulate whole-body homeostasis. The capacity of maintaining strength and generation of blood lineages lies within the skeletal system. Bone harbours blood and immune cells and their progenitors, and vascular cells provide several immune cell type niches. Blood vessels in bone are phenotypically and functionally diverse, with distinct capillary subtypes exhibiting striking changes with age. The bone vasculature has a special impact on osteogenesis and haematopoiesis, and dysregulation of the vasculature is associated with diverse blood and bone diseases. Ageing is associated with perturbed haematopoiesis, loss of osteogenesis, increased adipogenesis and diminished immune response and immune cell production. Endothelial and perivascular cells impact immune cell production and play a crucial role during inflammation. Here, we discuss normal and maladapted vascular niches in bone during development, homeostasis, ageing and bone diseases such as rheumatoid arthritis and osteoarthritis. Further, we discuss the role of vascular niches during bone malignancy.

## Introduction

The development of the skeletal system can progress *via* intramembranous ossification or endochondral ossification. During intramembranous ossification, MSCs (Mesenchymal Stem Cells) directly differentiate into osteoblasts to support bone growth. Endochondral ossification is, however, used to generate most of the bones in the skeletal system and occurs *via* the formation of a cartilage scaffold which is later replaced with bone through invasion of osteoclasts and osteoprogenitors (1). Invasion of osteoclasts and osteoprogenitors is mediated through the ingrowth of new blood vessels by the release of proangiogenic factors such as VEGF-A (Vascular Endothelial Growth Factor A). During the later stage of endochondral ossification, an extension of blood vessels towards the epiphysis facilitates the replacement of cartilage with trabecular bone and the formation of long bones (2).

Bone participates in many physiological mechanisms due to its high degree of plasticity, which is essential for maintenance of structure, protection and locomotion (3). Bone tissue consists of osteoblasts, bone lining cells and osteoclasts in addition to mineral deposits. Subsequently, it has a cavity filled with blood vessels and soft BM (Bone Marrow) (4). This part of the BM contains premature HSCs (Hematopoietic Stem Cells) and non-HSCs. Later these premature HSCs become mature, differentiate and get released into the vascular system. These cells interconnected with the vessel network in the BM and laid the foundation of ‘niche’, a dynamic environment for stem cells renewal and home to differentiated cells (5, 6).

Although bone cavity nurture HSCs, MSCs also takes part in cellular development and differentiation here. MSCs differentiate into adipocytes, pericytes and neuronal cells. These differentiations are termed as stromal network formation by a class of biologists (7, 8). Recent studies expand the knowledge of the heterogeneity of mesenchymal stem and progenitor components and their specific functions. These approaches allow us to understand their role in hematopoiesis and disease progression (9, 10). The BM contains multiple stem cell lineages, which participates in bone homeostasis and osteogenesis. These cells create a specialized local microenvironment, rich with growth factors and hormones. Due to the enriched microenvironment, the BM serves as a niche for metastatic cells, which disseminate from other organs to bone. The BM is considered a vibrant ecosystem that regulates tumour cells invasive, angiogenic and metastatic behaviour (11, 12). Incessant crosstalk between cells and vessels in the BM creates opportunities for the tumour cells to stabilize and interact with neural, mesenchymal and endothelial cells in the tissue microenvironment (13, 14).

Despite the aggressive approaches in detection and therapeutic interventions, tumour cell dissemination remains the foremost problem for cancer patients, particularly in bone metastasis. Metastatic dissemination is a well-controlled multistep process, which includes the crosstalk of tumour cells with the local microenvironment, especially within the vasculature. Human bone vasculature plays a key role in tumour progression and helps establish the secondary site for tumour development. The BM endothelial cells participate in homeostasis and help in maintaining bone integrity (15, 16). Different stress conditions modulate the bone vasculature and create a halt in blood flow in aged bones which affects bone density and BM homeostasis. Endothelial dysfunction also contributes to disease progression, especially in cardiovascular mortality.

## Diverse Vascular Niches and Perivascular Components of the Bone Marrow

The BM tissue microenvironment encompasses functional, cellular and non-cellular components including adipocytes, immune cells, pericytes and stroma (8, 17). The BM tissue is considered the most dynamic organ of the body due to its ability to create virtually all blood cell lineage throughout the entire life span of adult individuals (18, 19). The BM is an essential portion of the bone cavity to regulate bone homeostasis and facilitate the stem cell niche formation for self-renewal and differentiation of stem cells. Extensive studies have been piloted to probe the role of tissue microenvironment in homeostasis and disease progression, and interestingly, a major segment of the research ramble around the non-stromal cells. However, research signifying the role of stromal components in the fate of disease remains poorly explored. The term stromal cells is a vaguely-defined and consist of a network of neural, mesenchymal and endothelial cells with roles in homeostasis, tissue repair and diseases in every organ (20, 21). Immuno-oncologists largely term pericytes, diverse mesenchymal cells and endothelial cells as stromal cells, however, the term is loosely defined and used variably with and without the inclusion of vascular cells.

The BM stem cell niche is a very distinct site that is comprised of supporting cells and makes a promising microenvironment for cellular interactions and signalling (22, 23). The BM tissue is enriched with different cell lineages including hematopoietic and non-hematopoietic cells. All the non-stromal cells have their determined contribution in tissue development along with mesenchymal stem and progenitor cells. Interestingly, mesenchymal stem cells are being used interchangeably with mesenchymal stromal cells but a report of ISCT elaborates the differences between these two cell populations. Mesenchymal stem cells pose the ability of self-renewal and differentiation and mesenchymal stromal populations contain homing and immunomodulatory properties (24). To differentiate further, the mesenchymal stromal cells should show plastic adherence and express CD105, CD73 and CD90 markers and must not express endothelial and haematopoietic markers (25, 26).

The BM mesenchymal cells are precisely used in tissue engineering, tissue development and regeneration studies. Recently it has been observed that MSCs can be differentiated into ECs and VSMCs under mechanical stimulation (27). In another study, the combined effect of small molecule inhibitors of kinases and mechanical stimulation induces vascular cell-like phenotypic alterations in MSCs. Such inductions increase the expression of pericytes and endothelial markers *in-vitro* and also the regenerative abilities of MSCs (28, 29). On the other hand, the mesenchymal stromal network of the BM surrounds HSCs for regulatory crosstalk, which has essential relevant implications in stem cell biology and appeared as a principal regulator in bone metabolism (14, 30).

Nevertheless, the mesenchymal network is not limited to regulating the HSCs but encompasses the entire BM hematopoietic development and comprises lineage-specific differentiation, cellular trading, disease regulation and tissue structural maintenance (30, 31). As we posit that the vasculature is a completely different entity from the mesenchymal stromal components, a detailed investigation needs to be done to characterize each cell lineage. Recent technical progress expands the understanding of phenotypic characterization, anatomy, composition and unique functions of mesenchymal stromal components (32). These advancements allow us to understand the heterogeneity of the BM mesenchymal stromal components and how these multiple cell lineages orchestrate hematopoiesis and participate in malignancy (21). Interestingly mesenchymal stromal cells express similar cell surface markers as on activated ECs and mesenchymal stem cells (CD105+ CD45-). However, mesenchymal stromal cells only possess limited pluripotent potential with differentiation directed towards osteogenic, adipogenic and chondrogenic lineages, whereas mesenchymal stem cells also can regenerate ECs (24–26, 33). Bone marrow derived mesenchymal stem cells (CD105+, CD73+, CD90+, CD166+ and CD45-) cultured in VEGF rich medium show increased levels of endothelial-specific markers such as KDR and FLT-1 (34). Subsequently, Meng *et al.* shows the differentiation of mesenchymal stem cells into endothelial cells *in-vivo* (35). Nevertheless, mesenchymal cell are of increasing interest in regenerative medicine approaches to restore worn-out or damaged tissue.

## Diverse Vascular Niches in Bone Development and Homeostasis

Human skeleton organization is a highly dynamic system with a role in architectural support, homeostasis and blood cells formulation (36). Bone formation is a continuous process. The bone formation process in the course of early embryogenesis begins in two different modes, namely intramembranous and endochondral ossification, as discussed above (37). Pre-existing mesenchymal tissue transforms into bone tissue in both processes. Intramembranous ossification is the result of direct condensation of mesenchymal tissue in the bones. Skull, maxilla, clavicle and mostly the flat bones are the products of this process (38–40). In the mode of endochondral ossification, mesenchymal cells differentiate into intermediate cartilage, which is later replaced by bone. This process occurs in the femur and tibia, long bones of the system. Chondrocytes develop through mesenchymal aggregation during endochondral ossifications and help in the activation of osteoblast differentiation. Surrounding cells of chondrocytes formulate perichondrium, which has a quiescent state of cells and undergoes hypertrophy (41).

**Figure 1:**
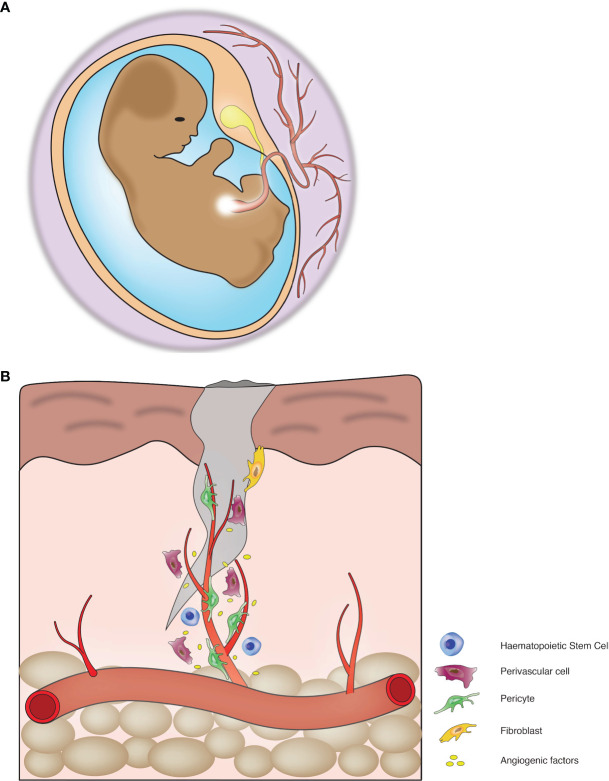
Blood vessels mediate tissue development and regeneration: **(A)** Blood vessels play crucial roles during organogenesis. **(B)** Blood vessels play a critical role in tissue repair and regeneration.

Recent developments in endothelial biology suggests that infiltration of vessels initiates bone formation during embryogenesis. During this process, endothelial cells vascularize the bone tissue and create a vascular bed throughout the length of the bones. The vascular bed is composed of countless capillaries, the central draining vein and arteries (42). Vascular infiltration into hypertrophic columnar cartilage is responsible for the generation of the primary ossification centre, which is eventually converted to a secondary centre during embryogenesis (36). The process of embryonic development includes vessel invasion to acquire nutrients and oxygen (**Figure 1A**). The vessel infiltration process during osteogenesis is somewhat similar to angiogenesis and directed by specialized structures in the vessels. These vessels are type-H and type-L; the distinction between these vessels is made up of comparative expression of endothelial markers, i.e., Endomucin (non-arterial vessel marker) and PECAM-1 or CD-31 (a canonical marker for endothelial cells). Type-H shows high expression, and type-L shows a low expression of these markers (43, 44). A high level of Endomucin and PECAM-1 is determined by Notch signalling, also responsible for higher expression of Kinase Insert Domain Receptor (a VEGF receptor). Blood vessel infiltration enables the enlistment of chondro-resorptive cells to disintegrate the existing cartilage and initiate osteoblastogenesis (45).

Ossification is a well-coordinated and regulated process, an essential part of homeostasis in the skeletal system. In general, bones contains three major compartments, i.e. epiphysis, diaphysis and metaphysis (46). Epiphysis contains the rounded portion of the growing end of the bones; diaphysis is the midsection of the bone, and metaphysis is the connection between epiphysis and diaphysis of the bones and is responsible for bone growth. Metaphysis contains an epiphysis growth plate and is compartmentalized in several zones based on the developmental process. Quiescent chondrocytes found in reserve zones divide rapidly in the proliferation zone and move to the epiphysis and begin to formulate hypertrophy in the hypertrophic zone. Few chondrocytes start calcification in the other zone, and the rest of the cells mature into the osteoblast and become a part of the development of the skeleton.

Embryonic osteogenesis is the outcome of the ossification process. Recent studies suggests the role of transcriptional regulation in the development of osteoblast. SOX9 is the major factor in endochondral ossification by controlling the development of the skeleton. SOX9 activates chondrogenic genes to initiate cartilage differentiation. Chondrogenic gene, i.e. *Col2a1* participates in craniofacial development and mutation in this gene can cause spondyloepimetaphyseal dysplasia. Loss of *Sox9* can hinder cartilage differentiation and lead to cell death ultimately (47–49). RUNX2 participates in skeletal development by regulating the genes in osteoblast differentiation i.e., *Spp1* and *Ibsp.* The latest outcomes suggests that deletion of *RUNX2* may lead to inhibition of osteoblast differentiation and loss of the above genes. Few studies reported that RUNX2 participates in immature osteoblast and hinder the maturation of osteoblast (50). OSX (Osterix) is another transcription factor that participates in the maturation of osteoblast and the generation of osteocytes. OSX regulates *Spp1* and *Sparc*, and inhibition of OXS resulted in irregular bone formation and accretion of abnormal cartilage (51).

Skeletal homeostasis is referred to as the dynamic balance of damage and repair of bone tissue. Bone formation and bone resorption are the two major processes of homeostasis facilitated by osteoblast and osteoclast, respectively (52). These cells are metabolically very active, and any irregularities may prime to numerous congenital disorders, deformities and bone sickness. In general, osteoblasts generate mineralized osteons, which are concealed in calcium deposition and later differentiate in osteocytes. After reaching a certain limit, osteocytes activate osteoclastic differentiation *via* RANKL, and when osteoclastogenesis leads to bone deformation, they secrete IGF to activate osteogenesis (53). Bone homeostasis is regulated by intrinsic and extrinsic factors such as mechanical stress, obesity, and senescence. A dynamic balance between osteoblast and osteoclast makes bone healthy and stronger. Recent studies show that sirtuin1 (SIRT1) participates in the differentiation of stem cells in the BM and bone-forming cells and regulate bone homeostasis. SIRT1 is a deacetylase and formulate epigenetic changes in histone or non-histone proteins (54). In bone, it is associated with bone mineralization. In mesenchymal stem cells, SIRT1 deacetylases β-catenin and prompts its nuclear localization, where it regulates osteogenic differentiation. It also regulates the differentiation of adipogenic tissue by inhibition of PPAγR2 (55). ATF4, a leucine zipper transcription factor activates *BGLAP2* in osteoblast and participates in terminal differentiation. Knockout studies show that it hinders bone homeostasis and osteoblast differentiation (56). AP-1, another transcriptional factor, which makes a complex with FOS, JUN and ATF, may act on osteoblastic enhancer gene and promotes osteoblastogenesis and regulate homeostasis (57, 58).

As discussed above, the vascular network is important for the development of the skeletal system; it plays a requisite role in homeostasis. Bone vasculature represents the prototypical hierarchical network of vessels and arteries, participates in paracrine signalling, blood perfusion, draining veins and interconnecting capillaries (59, 60). Here we have shown the specific role of vessel elongation required for the growth of cells during regeneration (**Figure 1B**). Long bone contains infiltrated vessels which are the source of blood, and some arteries invade the diaphysis and reach towards metaphysis near the growth plate. Type-H vessels are present in metaphysis and endosteum and are the major factor in regulating bone homeostasis. Type-H vessels are involved in the cross-talk between multiple cells in the bone and couple osteogenesis and angiogenesis (44). Hypoxia-inducible factors, especially HIF-1α, regulates type-H vessels. HIF-1α stimulates type-H vessel expansion, increases the number of osteoprogenitors and increase bone mass. Type-H vessels are also regulated by the Notch pathway. Any functional disruption in Notch signalling reduces the abundance of endothelial cells and type-H vessels in postnatal angiogenesis (61, 62).

Bone mineralization is a major factor in bone homeostasis. SOD3 (superoxide dismutase-3) regulates oxidative stress levels in cells by the formation of hydrogen peroxide from superoxide. The bone remodelling process involved resorption of the mineralized matrix through osteoclast and replaced through osteoblast by making new bones. Recent reports explored that SOD3**^-/-^** mice show reduced bone strength and impaired mineralization which affects bone mass and density (63). *Tang et al.* recently explored the Role of *Runx1* in osteogenesis and homeostasis. Runx1 binds to core-binding factor β (*cbfβ*) and form a heterodimeric complex to bind the promoter complexes. *Runx1* plays a key role in mesenchymal stem cells commitments for differentiation and regulates several signalling cascades involved in bone formation, especially WNT/catenin pathways which have a significant role in osteoblast-adipocytes lineage differentiation. This study shows that knockout of *Runx1* generates osteoporosis phenotypes in mice. Runx1 binds and regulate the expressions of *Bmp7* and *Atf4* promoters and participates in postnatal bone homeostasis (64). Vasculature plays a significant role in the development of bone and maintaining homeostasis.

The skeletal system is a highly important organ responsible for the maintenance of haematopoiesis and osteogenesis. This includes the production of hematopoietic stem cells and differentiation into mesenchymal stem cells, osteoblasts and other mature functional cells (65). Maintenance of stem and progenitor cells is crucial in terms of healthy whole-body homeostasis and function (66). The vessels in the bones supply oxygen and nutrients *via* the central nutrient artery, the periosteal artery or metaphyseal-epiphyseal artery. Blood flows *via* these vessels through a densely populated capillary network and drains through the central vein (67). Recent studies shows the crucial role of the BM vasculature in regulating the fate of stem and progenitor cells *via* BM niches (68, 69). The BM niches have a distinct microenvironment that is highly complex and predominantly consists of vascular components and signals responsible for regulating stem and progenitor cell survival, quiescence, mobilization, and differentiation (70, 71). These signals consist of cell surface ligands, soluble factors, or cell-cell interactions (68, 69).

As we discussed above the BM vascular niche consists of multiple endothelial cell subpopulations, namely type-L, type-H and arterial ECs (44). Subsequently, they are physically associated with osteoprogenitors. Functional differences between type-H and other BM vessels, and secretion of angiocrine factors, regulate blood cell proliferation and differentiation and therefore maintain homeostasis and function. Different from sinusoidal ECs, arterial ECs are found to be the major source of stem cell factor (SCF) in the BM, which is crucial for HSC function (72). Sinusoidal type-L vessels are mainly supported by LepR-expressing perivascular cells, which contribute to CXCL12-abundant reticular (CAR) cells that support HSCs and contribute to the adipocyte lineage (73–75). Distinct perivascular cell types from the mesenchymal origin are found to be important for the support of the specialized vascular niches (76, 77). Type-H vessels are covered with RUNX2 and Osterix expressing progenitors (44, 78–80). Subsequently, type-H capillaries and arterioles are associated with pericytes that express NG2 and PDGFR-β receptors together with Nestin expressing mesenchymal stem and progenitor cells (MSPCs) (81, 82). Arteriolar niches play a pivotal role in maintaining HSCs quiescence and HSCs distribution between the BM niches (81, 83) and HSCs prefer to localize within the different vascular niches in the BM. Imaging of HSCs localizing in the BM shows a highly abundant presence of both dividing and non-dividing HSCs in the central diaphyseal BM around sinusoidal vessels and distant from arteriolar vessels (84). A quiescent subset of HSCs was found to mainly localize around endosteal arteriolar vessels surrounded by NG2+ pericytes. Proliferative HSCs moved away from the arteriolar vessels towards LepR+ perisinusoidal vessels (81, 82). Thus, endothelial interaction with HSCs in the distinct BM vascular niches regulate HSC quiescence and proliferation. This interaction occurs mainly *via* the secretion of certain signalling factors by the BM ECs, which is critical for HSCs homeostasis. These factors consist of HIF-α, Notch ligands, CXCL12 and SCF (76, 85). Cellular crosstalk in the bone tissue microenvironment is operated through the vasculature. Ageing of vasculature has a specific role in the functional capacity of organs. Here we are discussing vascular ageing in the skeletal system in the next section.

## Ageing of Vascular Niches in Bone

Ageing is shown to affect the skeletal system *via* loss of mineralized bone and the increase of fracture risk and subsequently increases the risk of osteoporosis (86). In the process of ageing, the BM vasculature shows both morphological and metabolic changes with a significant reduction in arteriolar vessels. The reduction of type-H endothelium causes a decline in blood flow and reduced expression of angiocrine and pro-hematopoietic factors such as HIF-α, SCF, CXCL12 and Notch (76, 85). This decrease of angiocrine factors is often associated with poor angiogenesis, bone construction and increased risk of osteoporosis (87). HIF-α is a transcription factor that is responsible for the regulation of cellular response to oxygen levels (80, 88). In terms of bone angiogenesis, HIF-α expression is increased in ECs and osteoblasts under hypoxic conditions; this promotes the formation of new bone and the growth of new vessels *via* the expression of vascular endothelial growth factor-A (VEGF-A) and other proangiogenic factors (80, 89). In the metaphysis bone area, expression of HIF-α occurs in an oxygen-independent manner by type-H vessels (44). The endothelial decrease of HIF-α that is observed upon ageing, therefore, contributes to type-H vessel decline and a reduction of osteoprogenitors, osteogenesis and bone density (44). Interestingly, the presence of sinusoidal type-L vessels remained unchanged upon biological ageing (44, 90).

As mentioned in the paragraph before, endothelial signalling in the distinct BM vascular niches *via* Notch ligands, CXCL12 and SCF pathways regulate HSC homeostasis (76, 85). In aged mice, the BM ECs show significantly lower levels of these signalling pathways when compared to young mice (91, 92). Notch signalling is one of the most critical cell-cell interaction mechanisms that control cell fate (93). Notch activation in the BM leads to ECs proliferation and the formation of type-H vessels (94, 95). Subsequently, activation of Notch enhances HSCs and both PDGFR-β+ and NG2+ perivascular cells, indicating Notch as a mediator to promote vascular niche function (65, 76). CXCL12 is essential for HSC and lymphoid progenitor maintenance and quiescence (96). CXCL12 is expressed by BM EC, perivascular cells, osteoblasts, sympathetic neuronal cells, and Nestin+ perivascular stromal cells that are physically associated with HSCs (65, 70). Deletion of CXCL12 in ECs and MSPCs reduced HSC frequency and impaired long-term repopulation activity (75, 96, 97). Endothelial and perivascular SCF is crucial for HSC maintenance and survival (74, 98). SCF is expressed by perivascular stromal cells, arterial ECs, type-H ECs and sinusoidal ECs (65, 74, 76). Deletion of membrane-bound SCF causes the significant depletion of HSCs. Subsequently, depletion of SCF from peri-arterial mesenchymal stem cells also results in the depletion of HSCs, which indicates the importance of the mesenchymal compartment in HSC maintenance (74). Total HSC numbers increase upon ageing. However, age-related relocation of HSCs away from endosteal arteriolar niches correlates with a reduction of self-renewal and loss of quiescence (99–101). Subsequently, ageing of the BM vascular niche can induce this ageing-associated HSC phenotype. Infusion of young ECs is able to partially restore HSC function, suggesting a relationship between changes in the vascular niche and HSC ageing (85).

Ageing of the BM causes a set of complications leading to both haematological and non-haematological diseases. Haematological ageing inevitably leads to decreased functionality of the immune system, which comes with a range of complications. Impairment of the immune system increases the susceptibility for infection, autoimmune disorders, and haematological malignancies (102–104). Inflammatory responses by the immune system are an essential response to tissue injury and infection. Upon infection, ECs, MSCs and other hematopoietic and non-hematopoietic cells are activated. Activation of ECs leads to an upregulation of pro-inflammatory cytokines such as interleukins (IL) and TNF-α (105–107). In the BM this response stimulates HSC proliferation, migration, and differentiation to maintain the pool of immune cells (104). Inflammation changes both the morphology and function of the BM endothelium. These alterations show many similarities to changes that are observed in the aged BM niche (108, 109). Both inflammation and ageing induce myeloid differentiation and impair HSC self-renewal capacity (104). Subsequently, serum levels of pro-inflammatory cytokines such as IL-1, IL-6 and TNF-α are upregulated in aged individuals (110, 111). The presence of these pro-inflammatory cytokines also further enhances the myeloid skewing of HSCs (112). Other complications that can occur due to ageing of the BM are numerous cancers such as acute myeloid leukaemia or osteosarcoma and osteoporosis, which is also referred to as bone loss disease. Non-haematological diseases associated with skeletal ageing are OA (osteoarthritis) and RA (rheumatoid arthritis). These conditions are characterized by bone reabsorption of osteoclasts and high levels of pro-inflammatory cytokines such as IL-6, IL-11, and TNF-a (113, 114). Vascular ageing is one of the crucial aspects of the skeletal system to grow and differentiate. It has an impact on bone joint disease and bone angiogenesis. We have covered the inflammatory status of joint synovium in the coming section.

## Vascular Niches in Joints

Joints are built up by a series of different tissues that serve different individual functions. However, all tissues cooperate to maintain healthy joint movement and homeostasis. The diarthrodial joint is structured by the presence of muscle, bone, bursae, tendon, cartilage, joint capsule, synovial membrane, and synovial fluid.

The synovial cavity is surrounded by the joint capsule, which consists of fibrous connective tissue that is attached to both bones. The synovium, apart from diarthrodial joints, is also located in tendon sheets and bursae and is comprised of a surface layer of cells, referred to at the intima and subintima. Between all the intimal surface layers, fluid is located, which is high in hyaluronic acid and has non-adherent properties. The intima mainly contains bone-marrow-derived macrophages called type-A synoviocytes and fibroblast-like cells called type-B synoviocytes. Other than fibroblasts, B-synoviocytes express high levels of VCAM-1. The cells of the intima are responsible for the production of extracellular matrix molecules and mediation of synovial fluid clearance and production (115, 116). We have illustrated joint synovium in a healthy environment, which does not show any inflammation (**Figure 3**).

**Figure 2:**
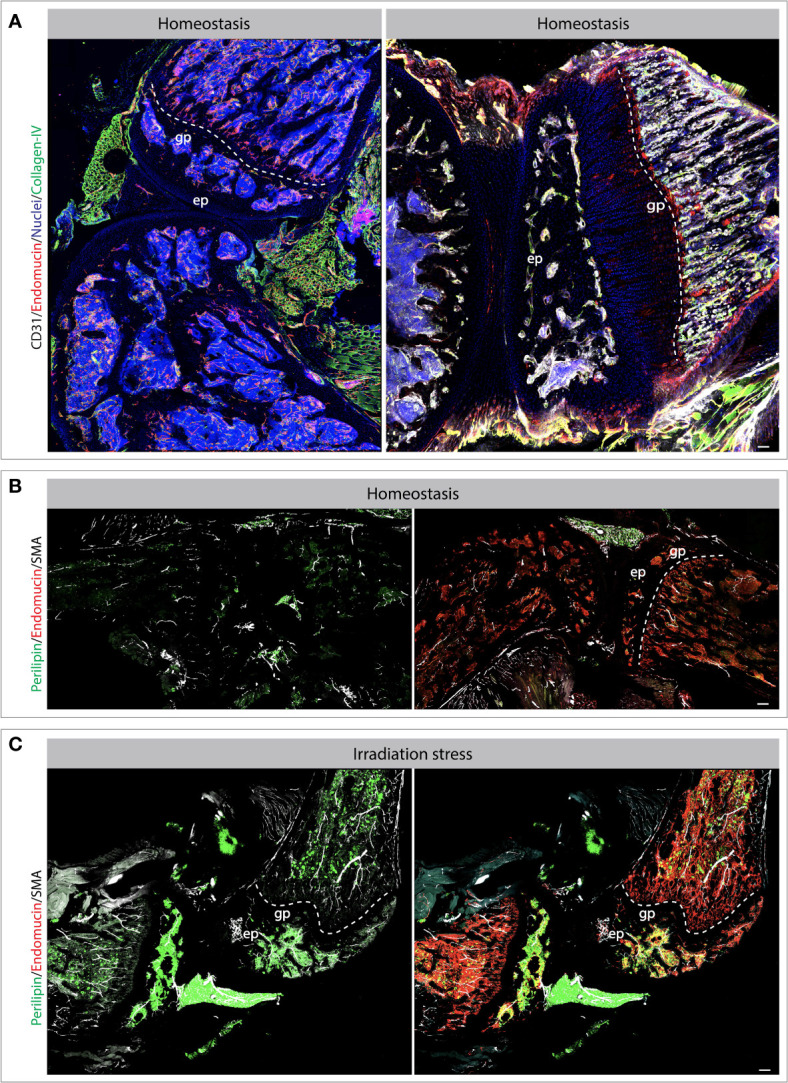
Bone and joint vasculature and perivascular niches. Confocal images showing blood vessels and perivascular cells in bones and joints. Exemplar bones and joint sections immuno-stained for endothelial and perivascular cell markers as indicated on the images **(A–C)**. The changes and vascular cells and perivascular microenvironments can be observed during stress conditions such as radiation-induced injury. Specifically, the tile view imaged demonstrate the changes in vascular morphology and accumulation of adipocytes post radiation **(B, C)**. Scale bar represents 200 *μ*m. gp, growth plate; ep, epiphysis.

Blood vessels and lymphatic vessels are located in a mostly collagenous tissue below the intima called the sub-intima (117). The synovium can be categorized in fibrous, areolar, and adipose depending on the composition of the sub-intimal layer. The sub-intimal layer of fibrous synovium is found in locations that are exposed to high pressure and are mainly composed of large collagen fibres (118) (**Figure 2A**). The areolar synovium has fewer collagen fibres, but more interfibrillar matrix and is found in places where the synovium moves freely over the joint capsule (118). Adipose synovium is found in intra-articular fat pads (119). These three different types of synovia can also be found together in a combination (118). Synovium is highly vascularized tissue with the presence of arterioles, capillaries and venules with fenestrae to supply oxygen and nutrients (**Figure 2A**). The distribution of vessels is organized in a non-uniform manner with the difference in population density according to the level of mechanical stress. Synovium that is subjected to higher levels of mechanical stress shows long loops of arterioles to supply more blood. However, the synovium that is subjected to very high mechanical forces has few vessels due to the low mechanical stress resistance of blood vessels. Capillary density is not only related to anatomical location but also the depth beneath the synovial surface. As previously mentioned, most blood vessels are located just below the intima, placing them in the sub-intima (120–122). In the synovial joint, VEGF, angiopoietin (Ang) and PDGF-*β* regulate vessel stability and induce fibroblast invasion. Complementary action of VEGF and Ang is essential for vessel formation, stability, and maturation; *via* regulation of EC proliferation, migration, survival, and pericytes/EC interaction. Dysregulated expression of VEGF and Ang in synovial tissue has been associated with multiple pathogenic outcomes such as rheumatoid arthritis. As shown, stress conditions alter vascular and perivascular microenvironments in the knee joint (**Figures 2B, C**). Osteoarthritis and Rheumatoid arthritis are the major chronic diseases associated with the joint. We have covered these two interesting aspects of chronic inflammation in the next section.

## Dysregulation of Vascular Niches in Osteoarthritis

OA (Osteoarthritis) is a degenerative and chronic joint disease, resulting from gradual degradation of articular cartilage and underlying bone remodelling. Articular cartilage is positioned on the joint surfaces and participates in movement (123, 124). Articular cartilage is subsequently made up of chondrocytes, collagen and proteoglycans, in a combined form of a hydrated extracellular matrix. It divides into several zones based on the distribution and alignment of chondrocytes and collagen fibres. During OA, subchondral bone shows sclerosis, abnormal vasculature and formation of osteophytes. It is also assumed that subchondral bone abnormalities contribute to cartilage disintegration (125). In disease progression, blood vessels invade the cartilages tissue and prompt the release of cytokines and create a low-grade inflammatory environment. This inflammatory environment hinders the identification of the molecular mechanism of OA initiation. Increased inflammation may induce angiogenesis and promote the invasion of vasculature in cartilage tissue. Studies suggest that vascular changes are the prime factors in the disease progression as it shows reduced perfusion. It is reported that subchondral bone has a link with cartilage and these interactions increase during the disease progression. Overexpression of TGF-β and osteoclast contributes to OA, and expression of TGF-β increases through osteoclast induced matrix resorption by positive feedback mechanisms. Targeting TGF-β can attenuate the OA by inhibiting aberrant bone remodelling and angiogenesis (126–128).

To explore the role of the vasculature in articular cartilage destruction, studies were conducted on temporomandibular joint osteoarthritis (TMJ OA). Vascular changes were analyzed with the expression of CD31**^+^** and α-SMA**^+^** in human and miniature pigs. They show that change in the vasculature is linked with the bone transformation from cartilage tissue. The study also shows the transformation of chondrocytes to osteoblast, confirmed with expression *COL2* and *RUNX2* in vascular stretches (129). In another study, a microfluidic-based *in-vitro* model presents the tissue vasculature role in OA progression. This cartilage on a chip method includes co-culture studies of primary endothelial cells with mesenchymal cell lines and investigates the osteogenic differentiation and tubes formation. Exposure of inflammatory cytokines to this model is able to attain the OA characteristics by depicting increased expression of MMP13 and ADAMTS5 (130).

The disintegration of cartilage tissue is completed by digestive enzymes. MMP and ADAMTS target their respective molecule i.e., collagen and aggrecan, respectively. Activation of these proteases is done by inflammatory cytokines secreted by chondrocytes, especially IL-1β and TNF-α (131). OA, instigates the functional and structural changes in bone and promotes catabolic protease activities. However, the role of inflammation in bone disintegration is debatable. Bone tissue undergoes plenty of modifications in OA by attaining a sclerotic phenotype. Such structural and molecular changes induced the BM lesions, which are sensitive to cardiovascular risk factors. These lesions make the way to cartilage damage and appreciate the subchondral bone changes (132). Despite the understanding of the clinical aspects of OA, the molecular basis is still vague. Recently, functional analysis of several genes explains the significance of molecular pathology. High throughput imaging analysis in mice resulted in 14 genes, and their functional role in the pathogenesis of OA and 6 out of them characterize for human pathogenesis. Based on rigorous screening, data highlighted 4 genes, namely *Bhlhe40, Pitx1, Sh3bp4 and Unk.* Reduced expression of PITX1 protein promotes subchondral bone thickness and is involved in OA pathogenesis. A detailed study in humans on gene expression shows that *Ccd6, Col4a2, Arhgap30, Gsdme, Unk, Josd1*plays a crucial role in the pathogenesis of OA (133).

Recently data shows a positive feedback mechanism that is present in bone-cartilage and vascular crosstalk. In the process of bone regeneration, type-H vessels participate in several mechanisms along with the coupling of osteogenesis and angiogenesis. This process involves type-H vessels, mTORC-1, chondrocytes and VEGF-A. This complex environment promotes activation of VEGF-A secretion and an increase in subchondral angiogenesis, resulting in OA (134). Targeting angiogenesis in the coupling of such pathways may affect the vascular invasion, blocking of VEGF induced angiogenesis shows a promising effect on cartilage destruction. Recent studies show that cytokine neutralizing antibodies are effective to show a potential effect on OA. It is observed that rapamycin can affect the pro-inflammatory cytokines i.e., IL-1. IL-6 and inhibits mTOR pathway, which may be an alternative approach to target OA in patients (135).

## Dysregulation of Vascular Niches in Rheumatoid Arthritis

Rheumatoid arthritis is a common chronic inflammatory autoimmune arthritis inclined by environmental and genetic factors and resulting in inflammatory pain in the hands, feet and knees. During the disease progression, angiogenesis promotes infiltration of inflammatory leukocytes and fibroblast into the joints and leads to bone destruction and hyperplasia in synovial joints. Hyperplastic conditions may prompt synovium to bone invasion and destruction, which is in line with the help of osteoclast cells. This process also inhibits the bone-forming process and leads to RA (125, 136).

Primary endothelial cells arranged in blood vessel lining and helped in cellular trafficking. Leukocytes migrate throughout the vessels and enter the connective tissue after interaction with the endothelial adhesion molecules. The endothelial lining of vessels activated by pro-inflammatory factors lead to the expression of adhesion receptors on the luminal side of endothelial cells and promote the binding of leukocytes and fibroblast. These interactions operate through ICAM-1, VCAM-1 and E-selectin majorly (137, 138). The landing of leukocytes and fibroblasts increases the inflammatory load and affects the joints. Leukocyte trafficking starts with interaction with selectin (CD15s) and is followed by the rolling on the endothelial surface *via* VCAM-1, which helps in transmigration. It has been observed that dendritic cells attract towards joint and secrete inflammatory cytokines and contributes to RA through IFN-α, IFN-β and IL-23. Dendritic cells regulate Th-cell response in RA and create an imbalance in the cytokine secretion and inflammation (139, 140).

**Figure 3:**
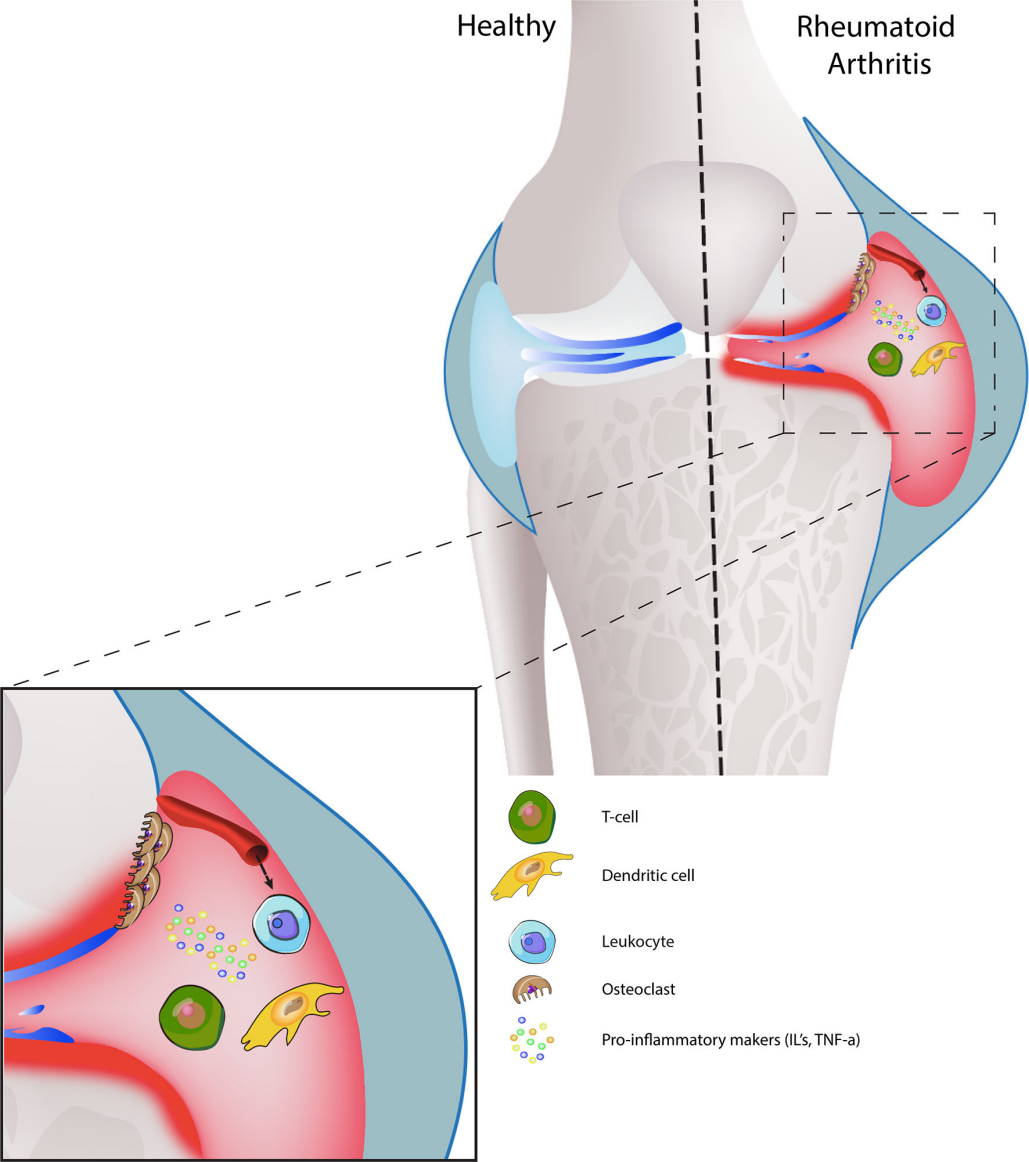
Joint synovium in healthy and diseased condition: Healthy joint synovium displays a thick layer of cartilage, medium synovial fluid levels in the joint cavity with intact synovial membrane and a strong, smooth outer bone layer (top left). In RA, the synovial membrane is swollen and has a high presence of synovial fluid, which leads to damaged bone and cartilage (top right). On the cell-interaction level, increased leukocyte infiltration promotes inflammation, hyperplasia, and bone destruction by osteoclasts. Dendritic-T cell interactions also release pro-inflammatory markers, which further enhances disease progression.

Vasculature changes contribute to the pathology of both conditions. In OA disrupted blood flow and ischemia in the subchondral bone reduce the nutrient supply to the articular cartilage, which lead to osteocyte cell death and articular damage (141). Subsequently, increased type-H vessel formation due to overexpression of VEGFA, PDFG-B and TGF-B induce pathological subchondral bone angiogenesis, therefore contributing to the development of OA (142–144). During RA, activated blood vessels expressing ICAM-1, VCAM-1 and E-selectin are responsible for leukocyte and fibroblast migration. Therefore, actively contributing to the progression of RA (145–148). The signalling process of joint inflammation, including the cellular cross-talk, depict the diseased conditions (**Figure 3**).

Recent reports suggest that the interaction of leukocytes with endothelial cells can increase after the TNF-α activation in endothelial cells. The level of TNF-α is found to be increased in RA pathogenesis. Few studies show that the generation of biologic DMARDs (Disease-Modifying anti-RA Drugs), which has specific targets in cytokine pathways, may affect the disease progression in RA patients (149, 150). Oxidative stress is one of the prime reasons for inflammatory activities in the joints. Interaction between immune cells and antigens create ROS in arthritis pathogenesis. Upregulation of p38 MAPK increases the ROS generation, which in turn induce the secretion of pro-inflammatory cytokines in RA. Activation of p38 contributes to cartilage damage, synovial inflammation and angiogenesis. Recent studies depict the importance of ROS inhibition in the prevention of RA (151, 152). Tissue vasculature has different properties in different organs. The specificity of each organ is supported through its specific vascular niches. We have explored this aspect of organ-specific vasculature in the following section.

## Tissue-Specific Vascular Niches and Vascular Changes

The kidney is an important organ in the health and homeostasis of the human organism due to its role in clearing the blood of toxins and waste products while maintaining haematological homeostasis *via* regulation of acid-base balance, red blood cell count and blood pressure *via* the secretion of hormones (153). The renal vasculature is highly complex and essential for renal function. Renal endothelial cells (RECs) show functional differences according to their location (154, 155). RECs can be subdivided into glomerular RECs (gRECs), medullar RECs (mRECs) and cortical RECs (cRECs). Each subtype of RECs differs from supporting the function of the renal gland. For instance, cRECs are surrounded by pericytes and smooth muscle cells, which can regulate the glomerular filtration rate (GFR) in the cortex. gRECs, on the other hand, are fenestrated to allow water passage yet restrict the passage of high-molecular-weight substances (156, 157). ScRNA-seq exposed even higher levels of heterogeneity across the RECs population with five sub-populations found in the gRECs population, nine for the cRECs population and ten for the mRECs (158). The heterogeneity of the renal vascular system may hold the potential to better comprehend and identify targets for a variety of renal conditions and disease such as chronic kidney disease (CKD). CKD is considered a major global problem, with 850 million people affected (159). Early-stage CKD already shows clear evidence of endothelial dysfunction, affecting vascular permeability, angiogenesis, inflammatory response, and immunity. Further endothelial damage leads to atherosclerosis and worsening of CKD disease prognosis with progression towards end-stage kidney disease (160, 161).

The lung has a high population of ECs that serve to maintain lung homeostasis. The vasculature of the lung can be subdivided into systemic bronchial and pulmonary circulatory systems. Quite remarkable of the lung vasculature is its capability to recruit available vessels to allow for an increase in flow with little increase of pressure during, for instance, exercise (162). The pulmonary ECs (PECs) play a key role in regulating oxygen exchange, controlling barrier function, and regulation of vascular tone *via* nitric oxide, serotonin, endothelin, and prostacyclin pathways (163). Extracellular interaction of PECs with circulating and surrounding cells is essential to maintain homeostasis by controlling thrombosis, inflammatory cell adhesion, angiogenesis, and vascular wall integrity (164, 165). Single-cell transcriptomics data of lung ECs show enrichment for immune, regulatory signatures, suggesting a role in immune surveillance. Subsequently, veins in the lung tend to have upregulated expression levels of genes involved in cAMP metabolism (166). cAMP is involved in controlling ciliary beat frequency and suppression of the pro-inflammatory activity of immune and inflammatory cells (167). Alterations of the pulmonary endothelium are involved in the pathogenesis of multiple lung diseases such as chronic obstructive pulmonary disease (COPD) or acute respiratory distress syndrome (ARDS). Lung diseases associated with pulmonary endothelial are mainly characterized by increased permeability leading to vascular leakage and oedema formation, the altered balance between vasocontraction and vasodilation, acquisition of pro-inflammatory phenotype, acquisition of pro-thrombotic phenotype and miscommunication with adjacent vascular cell wall (165, 168).

The liver is crucial for the maintenance of homeostasis due to its involvement in detoxification, immunity, metabolism, and nutrient storage. In order to fulfil these tasks, the liver is comprised of numerous different cell types apart from parenchymal hepatocytes. The non-parenchymal cells (NPCs) consist of liver sinusoidal endothelial cells (LSECs), Kupffer cells (KCs) and stellate cells (169). The nutrient-rich blood from the hepatic portal vein and oxygen-rich blood from the hepatic artery meet in the sinusoidal blood vessels (170). During this process, the LSECs of the sinusoidal vessels assist in clearing macromolecular waste and regulating hepatic vascularity (171). Other than most endothelial cells, LSECs possess a higher endocytic ability. As an example, 45% of all pinocytic vesicles are attributed to LSECs and LSECs are shown to be more efficient in absorbing/internalizing circulating antigens than dendritic cells or macrophages of the spleen and Kupffer cells and dendritic cells of the liver (172–174). LSECs are able to filter the blood *via* selective exchange of molecules in the blood and underlying stellate and hepatocytes due to their fenestrated morphology (175). Due to the lack of a basement membrane or basal lamina, there is direct access to the space of Disse (perisinusoidal space) for interaction between blood and hepatocyte or stellate cells (176). The LECs fenestrae have the ability to change their diameter according to as a response to the cellular microenvironment (177). The fenestrae are maintained by cooperative paracrine and autocrine signalling of hepatocytes and stellate cells. VEGF, NO and serotonin pathways have been shown to be involved in the maintenance and regulation of contraction or dilation of the fenestrae (178, 179). Loss or reduction fenestrae number in LSECs is referred to as defenestration (177). Defenestration leads to reduced hepatic uptake of lipoproteins which can cause hypolipoproteinemia (180). Subsequently, defenestration is involved in multiple disorders like liver fibrosis, atherosclerosis, or non-alcoholic fatty liver disease (NAFLD).

Malignancies in the kidney, lung and liver are associated with a poor prognosis due to their fast progression and metastasis. The role of vasculature is very important for the progression of metastasis from different organs towards the bone. We have created a dedicated section for bone metastasis and tumour dormancy. Across these organs, metastasis to the skeletal system is found to occur commonly (181–185). After initial tumour growth in the organ of origin, tumours cells undergo changes in cell signalling and cell-cell interactions such as reduced intercellular adhesion (186, 187). This causes the release of tumour cells in the circulatory system (188, 189). In most tissues, blood vessels only express adhesion markers such as VCAM-1 and ICAM-1 during exposure to inflammatory cytokines (190). However, the blood vessel ECs in the metaphysis of the long bones are characterized by their continuous expression of these adhesive proteins, which promote the interaction between circulating tumour cells (CTCs) (190, 191). The presence of voluminous sinusoids reduces blood flow in the blood vessels of the metaphysis, which allows for easier docking of CTCs (192, 193). Subsequently, the BM ECs release high levels of growth factors that attract metastatic tumour cells (194). Thus, across organ vasculature supports bone metastasis by its structural, cell-interaction and growth factor releasing characteristics.

**Figure 4:**
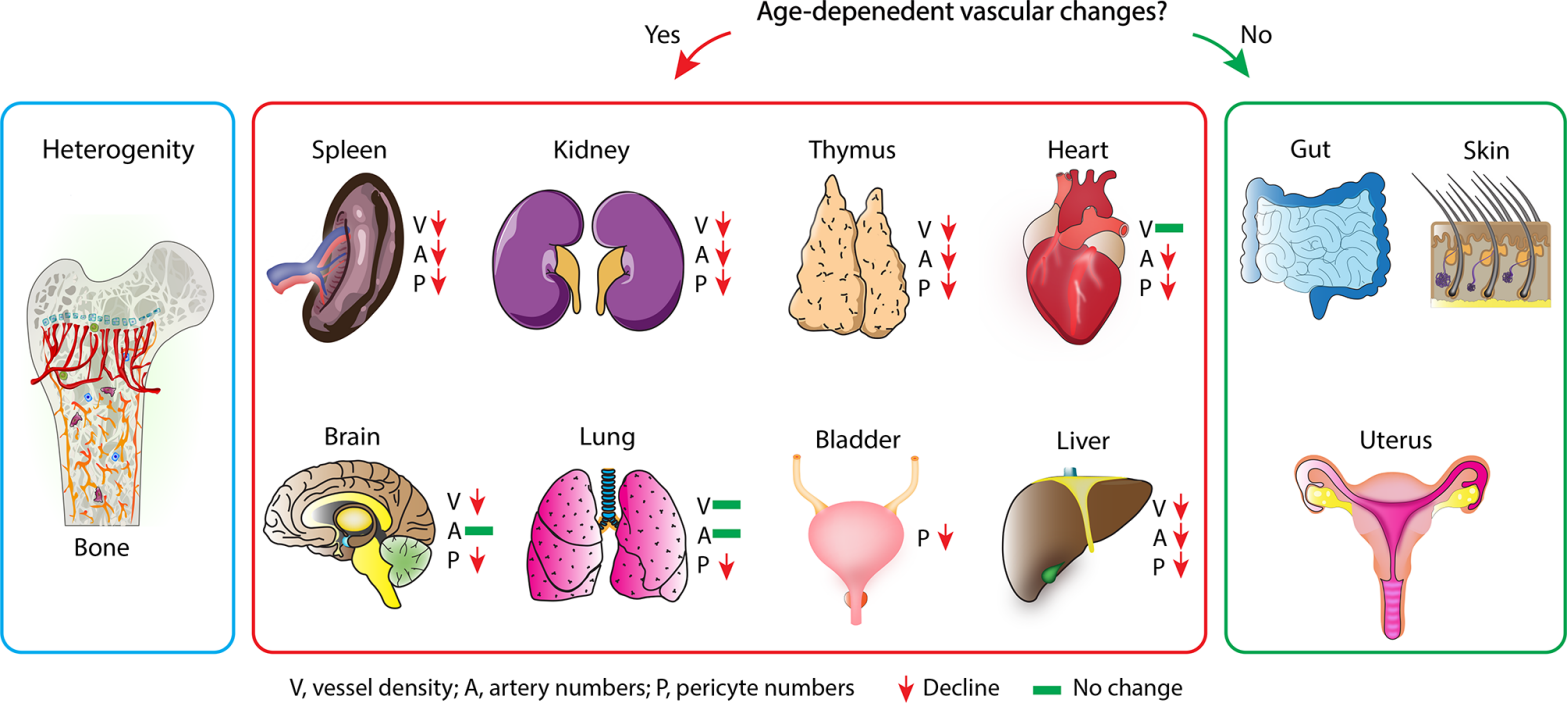
Vascular ageing across different tissues: Vascular ageing causes the decrease in endothelial heterogeneity of the bone but not the overall vessel density. This age-dependent change of the bone marrow vasculature is hallmarked by the decrease of type-H vessels (Left). Across all organs, different vascular changes are observed during the ageing process. Decreased vessel density, artery numbers and pericytes number are observed in spleen, kidney, thymus and liver. Fewer changes are observed in heart, brain, and lung (middle). On the other hand, gut, skin, and uterus maintain their vasculature integrity during ageing (right).

**Figure 5:**
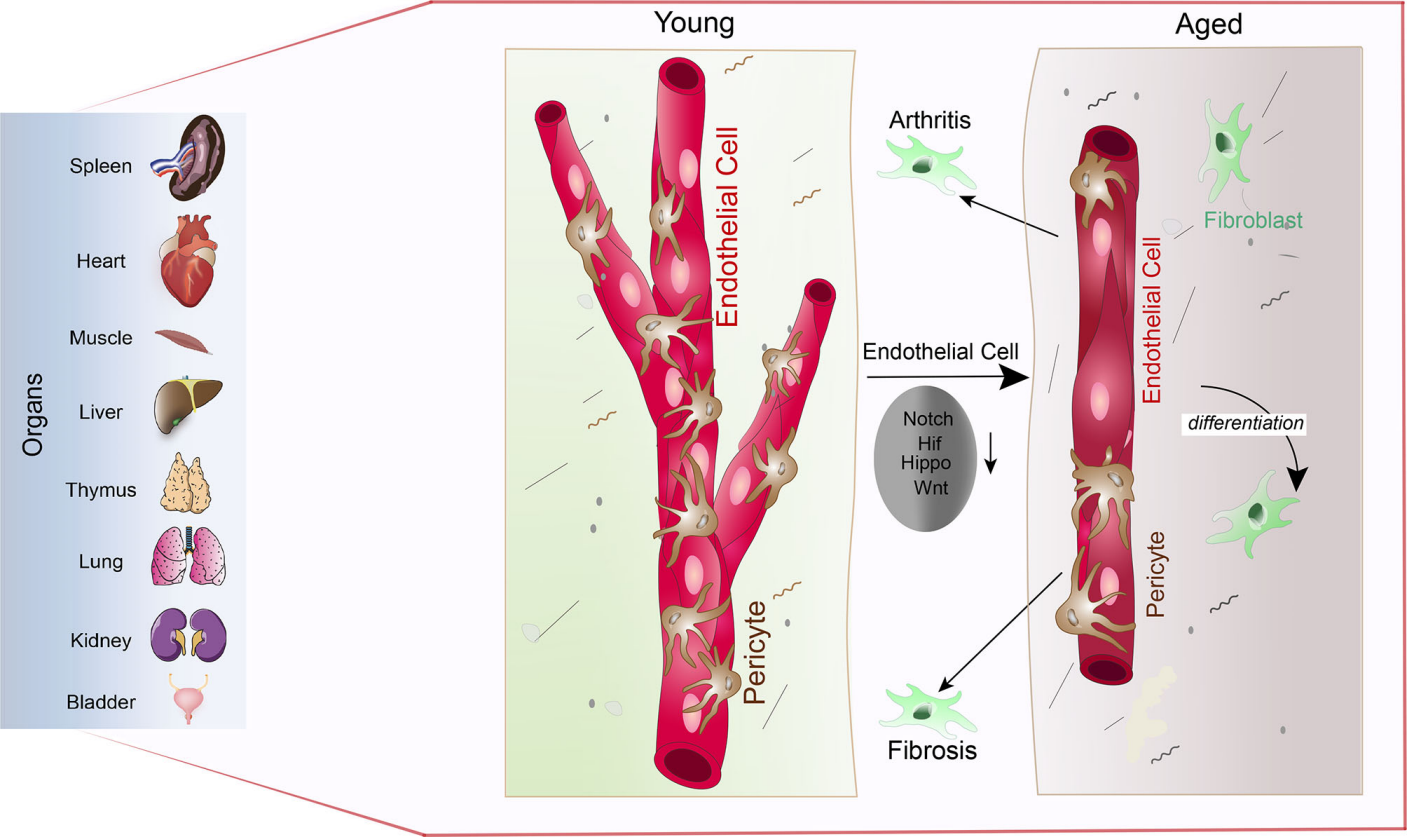
Vascular attrition is a primary hallmark of ageing: Pericytes abundantly cover the endothelial lining throughout the spleen, heart, muscle, liver, thymus, lung, kidney, and bladder (left) in young mice (young). Upon ageing, reduced Notch, Hif, Hippo and Wnt signalling in endothelial cells leads to pericyte loss *via* pericyte to fibroblast differentiation (aged). Loss of pericytes and accumulation of fibroblasts subsequently supports arthritic and fibrotic processes.

As described in the previous paragraphs, age-dependent changes of the vascular niche led to the loss of functional HSCs and osteoprogenitors. In ageing vasculature, inflammation, endothelial senescence, elevated oxidative stress, mitochondrial dysfunction, impairment of proteostasis and genomic instability is observed (195). Therefore, signalling from the microenvironment is an essential driver of stem cell and tissue ageing. Exposing the age-dependent changes of the vasculature has therefore has the potential to identify markers and targets of the ageing process across different organs and tissues. In recent years the topic of ECs heterogeneity across organs and tissues has become more of interest (166, 196, 197). Mapping of the tissue wide distribution of ECs, pericytes, mesenchymal stromal cells, and the matrix is essential to understand the age-related changes in the tissue microenvironment. Recently, loss of both vessel density and pericytes are exposed as a mark of ageing across tissue and organs (198). Ageing in organs show the specific role of vasculature (**Figure 4**) Tissues like the skin, gut and uterus who have high remodelling and regenerative capabilities (199–201) are, however, able to maintain the abundance of blood vessels and pericytes upon ageing (198). A similar phenomenon is observed in bones where the vessel density is unaffected by ageing (44). This can be explained by the relatively high regenerative capacity of bone when compared with the kidney, spleen, heart, or brain. Increased pericyte to fibroblast differentiation is observed with ageing, which could help explain the general loss of pericytes. Fibroblasts involved in joint inflammation and organ fibrosis are subsequently shown to originate from pericytes, wherein in the case of organ fibrosis, differentiated pericytes are considered a driver of fibrosis. Down-regulation of multiple signalling pathways responsible for the regulation of blood vessel maintenance and remodelling across multiple organs results in vascular attrition and the pro-inflammatory nature of ECs that is observed during ageing (**Figure 5**) (198). It is proposed that EC inflammation combined with alteration in the signalling pathways responsible for the regulation of blood vessel maintenance and remodelling, ultimately lead to loss of vasculature and accumulation of fibroblasts. Accumulation of fibroblasts *via* pericytes to fibroblast transition is known to occur in tumours and promote tumour growth and metastasis (202). To understand the details of metastasis in the bone microenvironment, we have dedicated a specific section. This will describe the tumour metastasis and dormancy in the bone microenvironment.

## Role of Vascular Niches During Bone Malignancy

Cancer metastasis is a distinctive mechanism of malignancy, and the invasion of bone is the most common choice of solid tumours, especially in the breast and prostate. Recent studies formulated that bone metastasis is the major cause of death in solid tumours (203, 204). Uprising clinical challenges during the dissemination lead to hyper mortality in patients. Prior to dissemination, tumour cells transition to mesenchymal to initiate the invasion through basal lamina and sustain in the circulatory system with the help of platelets. Tumour cells trigger platelets aggregation by binding to VWF (von Willebrand factor) and stimulating the VEGF secretion to support angiogenesis. Platelets thus secrete cytokines like LPA (Lipopolysaccharide) and make the molecular switch which contributes to tumour cell detachment and circulation in the bloodstream (205, 206). The mesenchymal transition of tumour cells is modulated with a few specific signalling cascades e.g., TGF-β and MAPK, and firmly associated with metastasis progression. In tumour cells, MAPK upregulation promotes MMP activation and, therefore, digestion of surrounding tissue and invasion of the tumour cells. Upregulation of MAPK shoves metastasis in a multidirectional way by activation of MMP, regulation of adhesive components and inhibition of retinoblastoma phosphorylation. Heparin-binding epidermal growth factor receptor upregulates the metastasis signalling *via* activation of MAPK in prostate cancer (207, 208). In addition to MAPK, NF-kB also participates in tumour metastasis *via* induction of EMT in tumour cells. In general, NF-kB signalling is regulated by an inhibitor of NF-kB (IkB). During cancer progression, the tumour necrosis factor-alpha receptor inhibits the IkB activity. IkB inhibition hinders the binding of NF-kB and IkB, which leads to hypoxic conditions and NF-kB mediated activation of HIF-1α. HIF-1α triggers the EMT in tumour cells and promotes metastasis (209, 210).

Although the reason for cancer metastasis is less implicit, the whole process is well coordinated. The tumour microenvironment is a major factor in metastasis as it contains multiple cell lineages, which interact with the tumour cells (211). Out of the mesenchymal stromal component, it is evident that macrophages participate in all the phases of the metastasis cascade. These macrophages are derived from monocytes and are involved in cancer progression, as explained in recent studies. Simultaneous deletion of IL-4 and CCR2 alone with monocytes add back approach, explained that bone tissue originated macrophages do not participate in tumour cells establishment in bone tissue microenvironment (11, 212).

During circulation, platelet interaction with tumour cells upregulates CCL2 expression on tumour cells and thus promotes vascular permeability. Tumour cells engaged with stromal cells *via* CCL5 metastasize and make a stable network at bone tissue (213). Bone vasculature is different from other organs due to fenestrated vessels architecture, which contributes to tumour engagement and extravasation by constitutive expression of adhesion proteins. Recent time-lapse imaging and single-cell sequencing studies show the tumour-specific blood endothelial cells, which helps in metastasis and are a part of cells that express *csf-1* (214). Type-L and type-H vessels express proteins like P-selectin, E-selectin, ICAM-1 and VCAM-1, which allow tumour cells to adhere and infiltrate to acquire extravasation (215, 216). During the extravasation, endothelial cells express and secrete growth factors, i.e. stromal cell-derived factor-1 (SDF-1), which promotes tumour cells to engage and establish a cell mass through their interactions with the BM cells (217).

Recent studies show the specific role of tumour-stromal cell interactions in cancer progression and regulation. CXCL12, also known as stromal cell-derived factor-1, and its localized receptors participate in cellular interaction during cancer progression and metastasis (218). CXCL12 and its receptors can induce multiple signalling processes that control gene transcription, cellular survival and apoptosis. The binding of CXCL12 and its receptor CXCR4 initiates the membrane changes and GTP exchange, which leads to dissociation of Gα units of G proteins. Dissociation of Gβ/Gγ activates phospholipase C (PLC-β) and induce the catalysis of PIP2 into IP3 and DAG signalling and chemotaxis. Interaction between CXCL12/CXCR4 initiates the phosphorylation of CXCR4, which supports the calcium flux and activation of PI3K, MAPK signalling and thus induce cancer cell proliferation (219–221).

Interestingly, CXCL12 secretion through osteocytes functions as a chemoattractant and helps in homing and retention of CXCR4 expressed cancer cells in the BM microenvironment. Tumour cell homing requires interactions with the ECM, with a crucial role for integrins. Integrin expression on tumour cells mediate critical interaction in tumour development. Integrin α2β1 expressed on tumour cells binds with type-I collagen, utmost ample protein available in bone. Studies show that binding of integrin with collagen type-I activates the integrin associated kinases and induce cytoskeleton rearrangement through activation of Rho pathway. Activation of RhoC GTPase is known to be a prominent factor for metastasis, and it helps in the invasiveness of tumour cells and metastasis to bone (222, 223). Recently, it is suggested that once the tumour cell attains bone proximity, plenty of them undergo apoptosis, and few of them survive. Tumour cells bump into the unreceptive milieu, which hinders the instant tumour growth in the bone environment. The BM niche produce multiple factors like Annexin A2, TGF-β, CXCL12 and IGF (insulin-like growth factors), which allows tumour cells to remain in the dormant stage (223, 224). Studies observed that tumour cells express a high level of Axl during dormancy. Axl controls cellular proliferation, EMT and innate immune response in general. Osteoblasts secrete GAS 6 and activate Axl receptors in tumour cells. Co-culture studies of tumour cells with pre-osteoblastic cells show that osteoblasts reduced the proliferation of tumour cells, which can be regulated by low expression of Axl *via* targeting of TGF-β and TGFBR2. The expression of TGF-β and TGFBR2 is elevated in co-culture studies and thus contributes to tumour cell dormancy. The BM vasculature niche provides stability to tumour cells and supports the tumour dormancy due to the low sinusoidal blood flow and large vessel diameter (225).

Dormancy of tumour cells depicts the progression stages of cell cycles, i.e. G0/G1. Such cells remain dormant for many years until activation occurs. Dormant tumour cells show higher expression of p38 MAPK signalling and downregulation of ERK MAPK signalling pathways. It is reported that p38 controls the grid of quiescent transcription factors, responsible for cellular growth/arrest and self-renewal genes. TGF-β also contributes to cancer cell dormancy *via* the regulation of activation of p38 signalling and quiescence. These specific regulators in disseminated tumour cells marks as a dormant signature in cancer cells. In addition to the predominance of p38 activity, NR2F1 also regulates tumour cell dormancy. NR2F1 is a nuclear hormone receptor that regulates induced pluripotent reprogramming and also neural cell crest differentiation. NR2F1 arrest the cell growth in disseminated tumour cells *via* the regulation of SOX9, NANOG, SOX2 and RARβ (226–229).

Disseminated tumour cells get support from osteocytes and start a positive feedback mechanism that initiates differentiation of osteocytes in osteoblast and/or osteoclast. Tumour cells recognize secretary molecules i.e. CCL5 and CXCL12 released from stem cells and osteocytes (230). Due to the positive feedback mechanism, this recognition prompts the osteocytes to release growth derived factors (GDF 10 & 15) and secretion of PTHrP (parathyroid related hormone protein) from tumour cells. PTHrP has a specific receptor PTH1R on the surface of osteoblast cells (231). After ligand interaction with the receptor, osteoblasts secrete RANKL (receptor activator of NF-KB ligand). RANKL binds on the RANKL receptor on osteoclast and induces osteoclastogenesis (232). Recently it has been found that EZH2, a transcriptional factor, play a significant role in bone metastasis. EZH2 promotes PTHrP expression *via* integrin β1 and the knockout of *EZH2* inhibit breast cancer-induced bone metastasis (233). However, RUNX2, a transcription factor, also participates in osteoclastogenesis. Phosphorylation of RUNX2 by integrin α_v_β_3/_sma5 cascade or integrin α_v_β_3/_src/rac1 cascade activates Akt pathway and leads to upregulate NF-kB expression, activation of RANKL, resulting in osteoclastogenesis (234–236).

This process induces bone resorption and secretion of growth factors from osteoclast cells. All the growth factors maintain the function of osteoblast and osteoclast, along with tumour proliferation. This process disturbs the homeostasis and lead to the formation of bone lesions and release the growth factors, which in turn promotes tumour growth and increase bone resorption. This feedback loop, named as “vicious cycle”, amplified the metastatic lesion formation in bone and ultimately progressed towards bone fracture and hypercalcemia (237, 238).

## Author Contributions

NK and PS wrote the review. All authors contributed to the article and approved the submitted version.

## Conflict of Interest

The authors declare that the research was conducted in the absence of any commercial or financial relationships that could be construed as a potential conflict of interest.

## Publisher’s Note

All claims expressed in this article are solely those of the authors and do not necessarily represent those of their affiliated organizations, or those of the publisher, the editors and the reviewers. Any product that may be evaluated in this article, or claim that may be made by its manufacturer, is not guaranteed or endorsed by the publisher.
